# Supplementary material for: Oncogenic herpesvirus KSHV triggers hallmarks of alternative lengthening of telomeres
Source: Nat Commun. 2021 Jan 21;12:512. doi: 10.1038/s41467-020-20819-4 (PMC7820467; doi:10.1038/s41467-020-20819-4)
Supplement: Supplementary file 1 — Supplementary Information [file 41467_2020_20819_MOESM1_ESM.pdf]

Supplementary information

Oncogenic herpesvirus KSHV triggers hallmarks of alternative lengthening of telomeres

[1,2]Timothy P. Lippert, [1]Paulina Marzec, [1]Aurora I. Idilli, [1]Grzegorz Sarek, [3]Mark Bower, [4]Paul J. Farrell, [4,5]Päivi M. Ojala, [2]Niklas Feldhahn* & [1]Simon J. Boulton*.

[1]The Francis Crick Institute, 1 Midland Road, London NW11AT, UK.

[2]Department of Immunology & Inflammation, Centre for Haematology, Du Cane Road, London W12 0NN, UK.

[3]National Centre for HIV Malignancy, Department of Oncology, Chelsea & Westminster Hospital, Fulham Road, London SW10 9NH, UK.

[4]Section of Virology, Department of Infectious Diseases, Imperial College London, Norfolk Place, London W2 1PG, UK.

[5]Translational Cancer Medicine Research Program, University of Helsinki, Haartmaninkatu 8, Helsinki 00290, Finland.

*Equal contribution

Correspondence: simon.boulton@crick.ac.uk

Supplementary figures

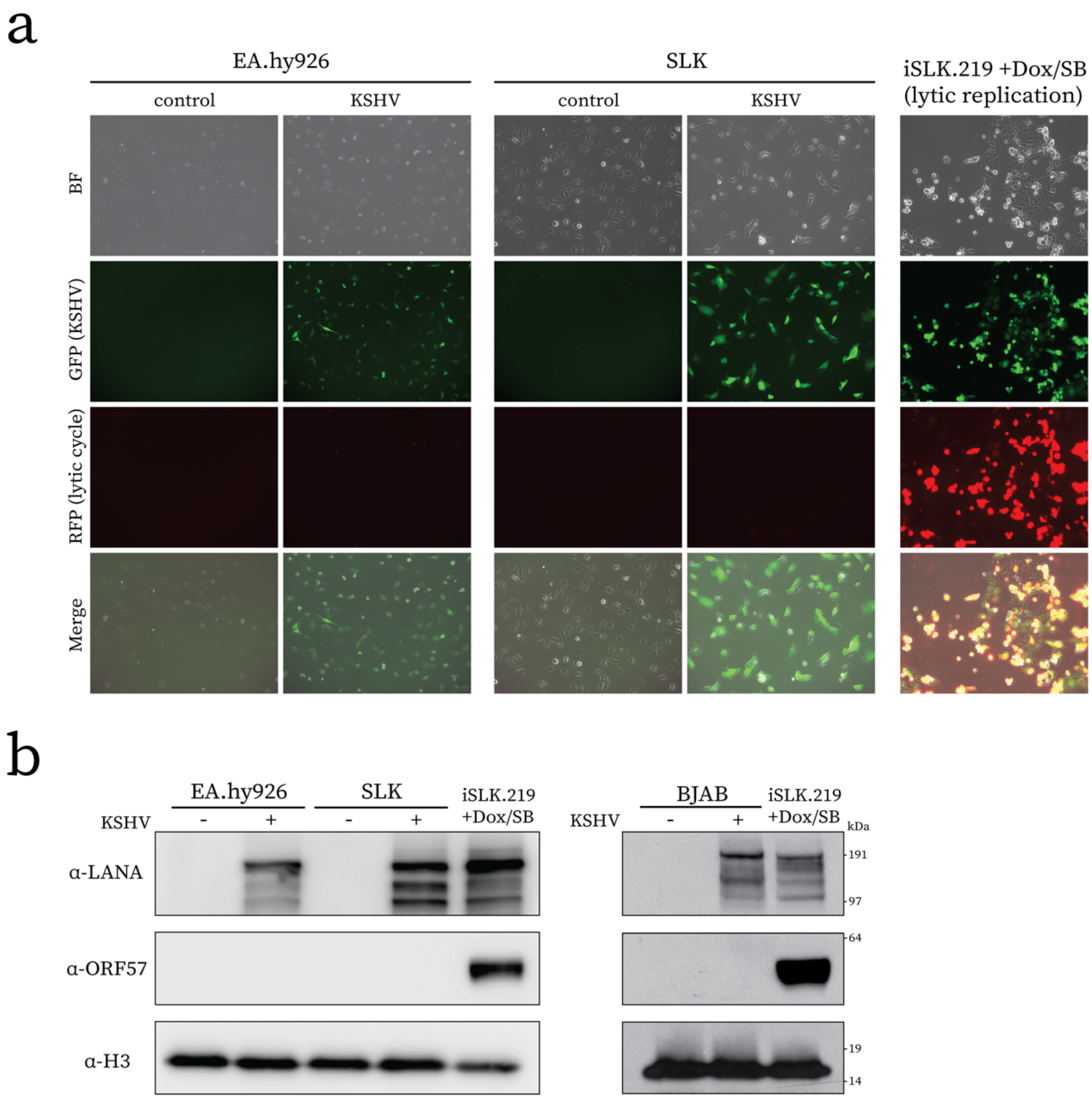

Supplementary figure 1: KSHV infection is strictly latent at the time point of analysis. a, Analysis of fluorescent markers encoded by rKSHV in cell lines included in the analysis. GFP expression indicates cells with KSHV episomes, RFP under control of main lytic promoter (details in methods section). iSLK.219 producer cells included as positive control for viral lytic reactivation. **b,** Representative Western blot of markers of viral life cycle. LANA as a proxy of viral infection and latency, ORF57 (viral mRNA processing factors) as marker for viral lytic reactivation, and H3 as loading control were blotted for. Image representative of result obtained from biological triplicate.

a

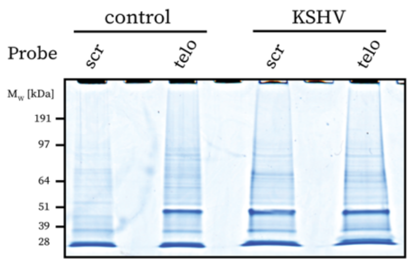

b

BJAB

	Protein	ΔUP control	ΔUP KSHV⁺
Resection/MMEJ	MRE11	30	81
	RAD50	36	160
	NBS1	14	69
	PARP1	11	24
	Lig1	2	nd
	Lig3	3	10
	XRCC1	nd	6
	Polθ	nd	nd
	CtIP	nd	nd
NHEJ	DNAPk	134	88
	Ku70	32	26
	Ku80	42	26
	Lig4	nd	nd
	XRCC4	nd	nd
	Artemis	nd	nd
Shelterin	POT1	28	96
	TRF1	29	92
	TRF2	58	138
	RAP1	35	109
	TIN2	35	123
	TPP1	nd	nd

BJAB

	Protein	ΔUP control	ΔUP KSHV⁺
ALT/HR	RPA1	12	41
	RPA2	1	5
	RPA3	nd	2
	RAD51C	1	1
	RAD52	nd	nd
	SLX4	6	51
	SLX4IP	1	17
	ERCC4	1	12
	FEN1	14	6
	NR2C2	2	14
	NR2C1	nd	1
	BLM	6	1
	ATRX	nd	nd
	DAXX	1	nd
	Sp100	1	nd

Supplementary figure 2: Results of spectral peak calling analysis. a, Material recovered from BJAB cells (control co-culture and KSHV-infected) PICh experiment subsequently processed by mass spectrometry was analysed by PAGE and colloidal blue staining. Mass spectrometry analysis was performed once and on the samples analysed on this gel. **b,** Table showing telomeric enrichment of DDR proteins at telomeres in BJAB cells upon infection. Values denote number of unique peptides enriched specifically at telomeres, ΔUP (unique peptides in scrambled control pulldown subtracted from unique peptides in telomere pulldown). Any key factors involved in pathways indicated, which were not detected in this experiment are indicated by nd (not detected). Source data are provided as a Source data file.

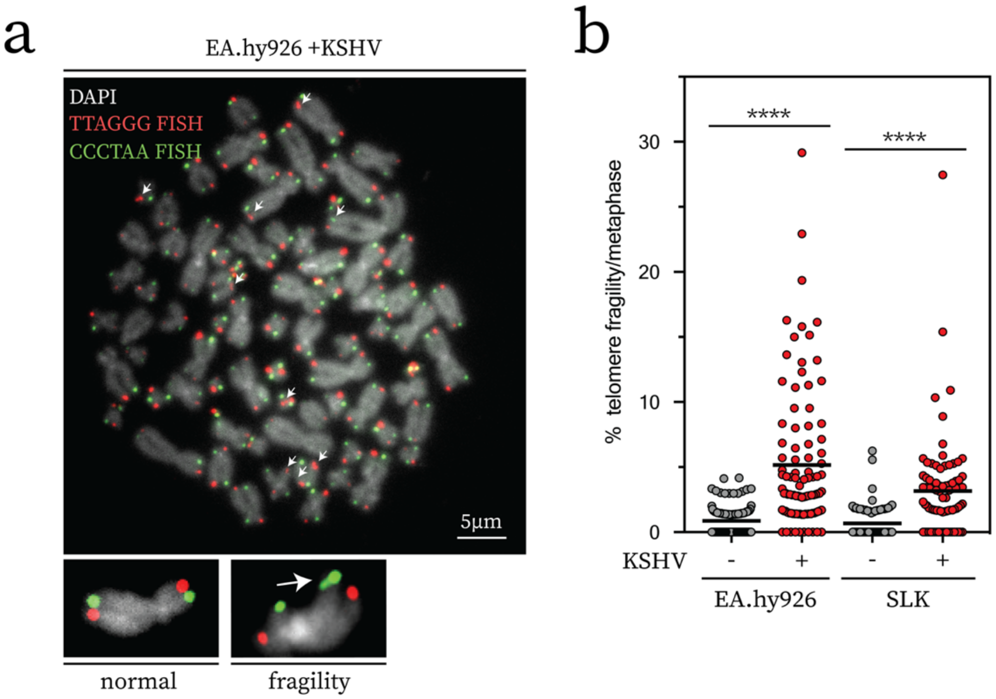

Supplementary figure 3: Cells harbouring latent KSHV show elevated telomere fragility. **a,** Representative metaphase CO-FISH micrograph of KSHV-infected cells. Magnified individual chromosomes shown below, with normal signal appearance (left) and smeared signal characteristic of telomere fragility (right). Throughout images, arrows denote incidences of telomere fragility as determined by analysis. **b,** Quantification of telomere fragility in cell lines. Total number of chromosomes and number of chromosomes with at least one fragile telomere were determined by blinded count and percentage chromosomes with fragile telomeres was plotted. Data was obtained from three independent biological replicates, at least 40 metaphases each. Mean differences were tested for statistical significance using an unpaired, two-tailed t-test (****$p<0.0001$ in both EA.hy926 and SLK cells).

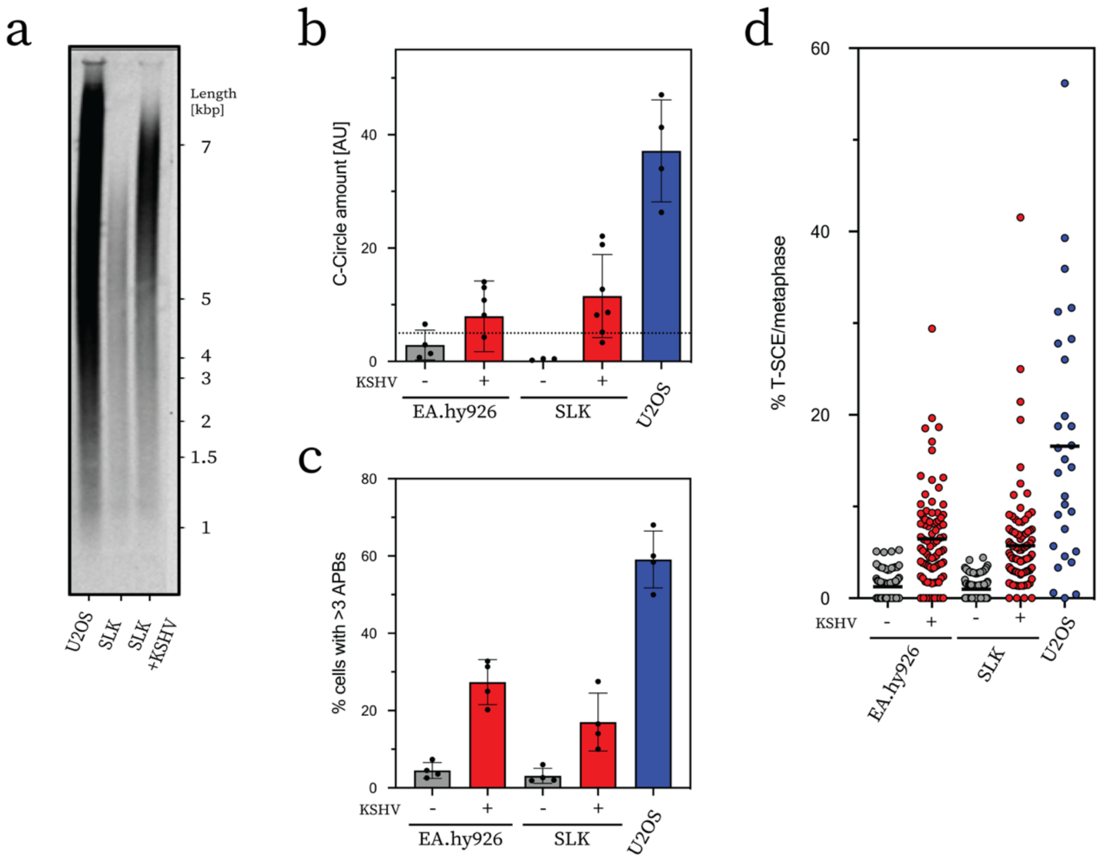

Supplementary figure 4: Absolute telomere length increase upon KSHV infection. Representative **a,** TRF Southern blot analysis, genomic DNA was loaded as indicated, separated by electrophoresis, and subjected to capillary transfer and hybridisation with probe specific to telomeric DNA. Blot representative of biological triplicate KSHV$^+$ and uninfected co-cultured cell lines as included in all other analysis, U2OS included here as positive control for ALT telomere length distribution. **b,** Comparison of C-circle induction in infected cells with level in U2OS cells, data representative of biological quadruplicate. **c,** Analysis of APBs in infected cells and U2OS, data representative of experimental triplicate. **d,** T-SCE incidence in comparison to level observed in U2OS cells, data representative of biological triplicate, except for U2OS, where it was performed in biological duplicate. All error bars represent SEM.

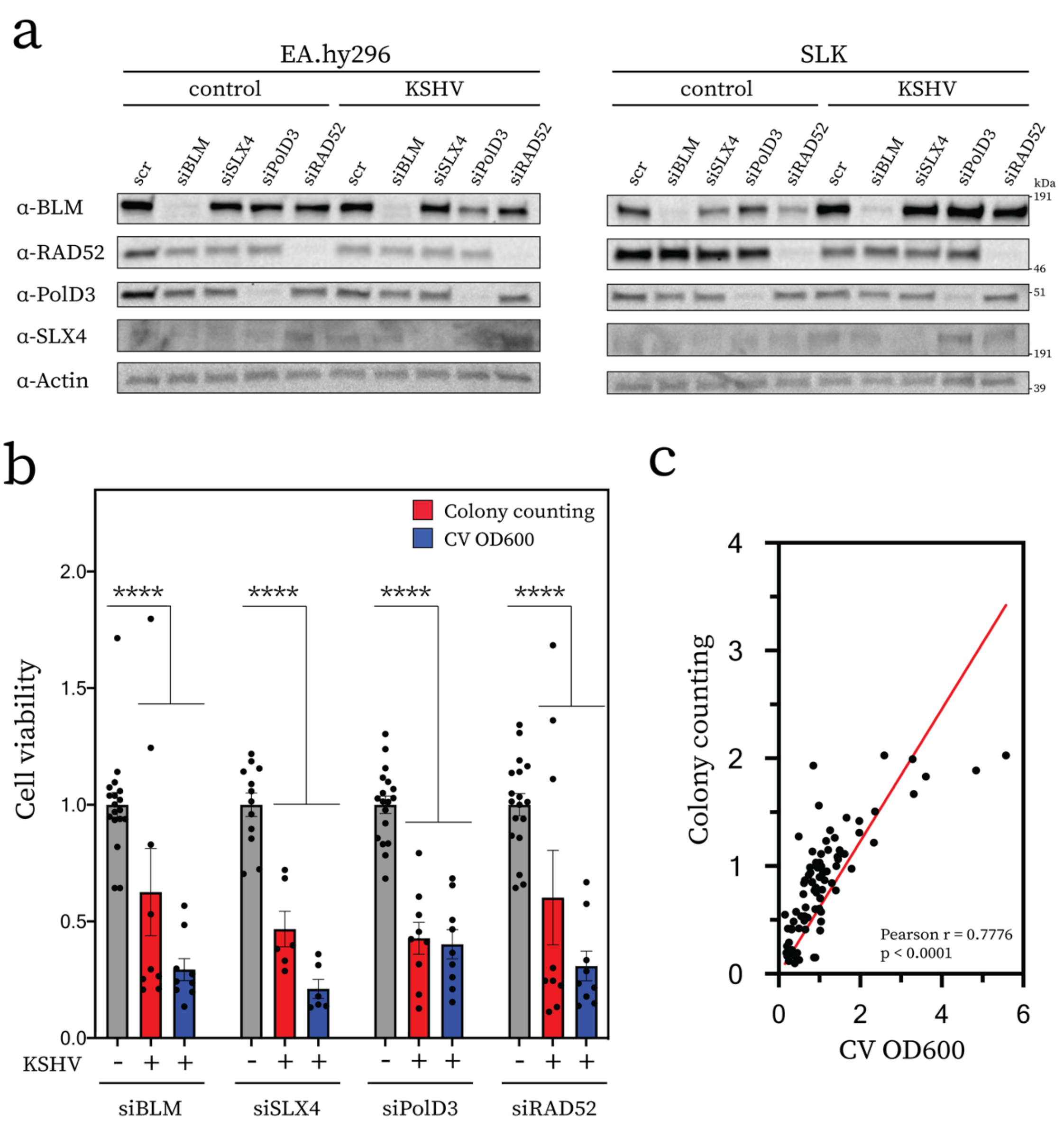

Supplementary figure 5: Analysis of cell proliferation upon knockdown of ALT factors. a, Western blot validation of knockdown efficiency in all cell lines examined. Blot representative of at least three independent experiments, knockdown efficiency was consistent across biological replicates throughout. **b.** Quantification of cell viability. Data presented as number of colonies or absorbance at 600nm relative to scrambled knockdown control in respective cell lines, and normalised to mean value of uninfected parental cell line (grey). Experiment was performed in biological triplicate using independently established cell lines, and in technical triplicate. Errors bars represent SEM, statistical significance was tested by one-way ANOVA (****p<0.0001 for all comparisons made). **c,** Correlation of analysis performed in **(b)**. Each dot represents one well as analysed by colony counting and photospectrometry, slope of line of best fit corresponds to Pearson's correlation coefficient as shown in graph inset.

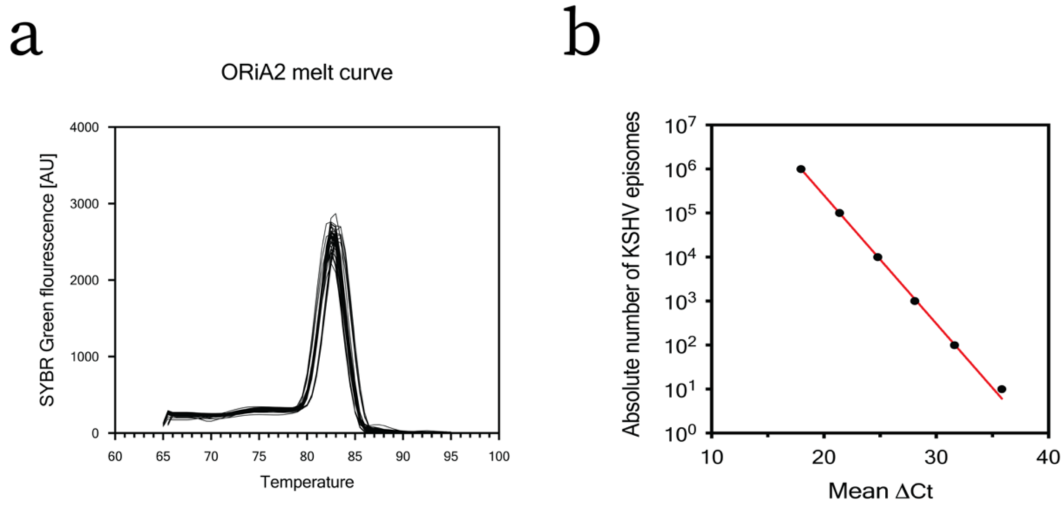

Supplementary figure 6: Summary of KSHV copy number analysis. **a,** Melt curve of product obtained from amplification using DNA from infected cells. **b,** Standard curve of fixed number of molecules amplified on the same plate as samples analysed in each experiment. Red line present semi-log line, which was used for interpolation.

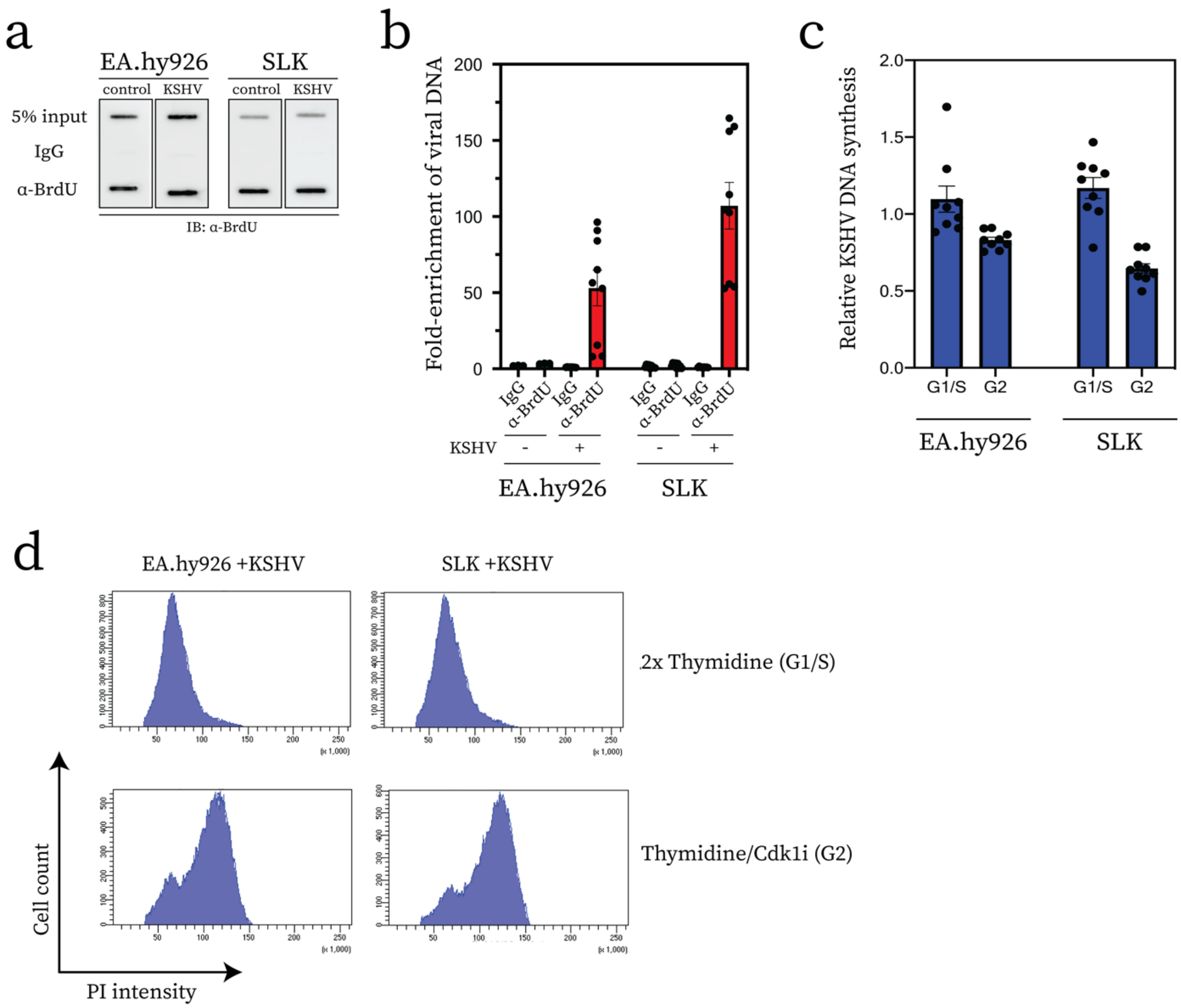

Supplementary figure 7: a, Representative blot showing BrdU incorporation and pulldown efficiencies in G2/M arrested cell lines as indicated. Similar levels of BrdU were pulled down specifically in each condition. Experiment performed at least three times with the exception of uninfected SLK cells, where it was only performed once. **b,** Quantification of qPCR signal obtained using equal input material for each pulldown and cell line shown below blots. Data representative of biological triplicate with exception of SLK uninfected control (one repeat), all data normalised to mean signal obtained from KSHV^{+} IgG control pulldown. **c,** Comparison of KSHV DNA synthesis during G2/M and S phase. Data from three independent biological replicates. KSHV DNA synthesis was determined by ddPCR measuring the number of KSHV amplicons relative to housekeeping control gene RPP30. Error bars represent SEM. **d,** Representative cell cycle profile of cells used for analysis presented in **(c).**

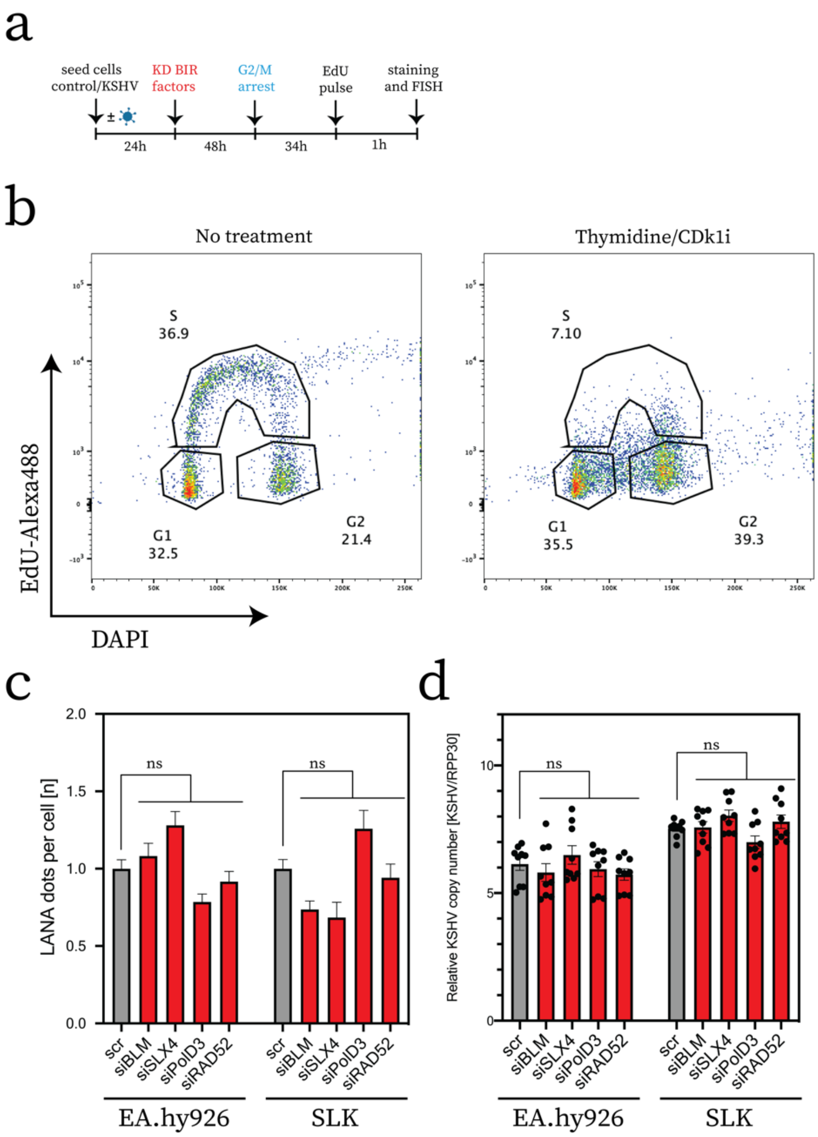

Supplementary figure 8: Control measurements for BIR foci analysis. a, Schematic overview of EdU Click-iT experiments. **b,** Representative dot plot of untreated (left panel) and double thymidine/Cdk1i (RO-3306) arrested cells (right panel). DNA content (DAPI) and DNA synthesis (EdU) was analysed by Click-iT chemistry and flow cytometry. Gates were drawn on fluorogram on the basis of populations apparent in untreated control, which allowed quantification of proportion of cells at each cell cycle stage. Plot representative of all cell lines tested. Dot plot displays measurements obtained from 200,000 cells for each condition. **c,** Relative number of LANA foci per nucleus for all cells included in BIR foci analysis. Data from all three biological replicates and presented as average normalised to scrambled knockdown control. **d,** Relative number of KSHV genomes per cell at time point of analysis as determined by ddPCR of input used for BrdU IP assays. Data representative of three independent biological replicates, performed each in technical triplicate. Error bars represent SEM, $^{ns}p>0.1$ as determined by one-way ANOVA.

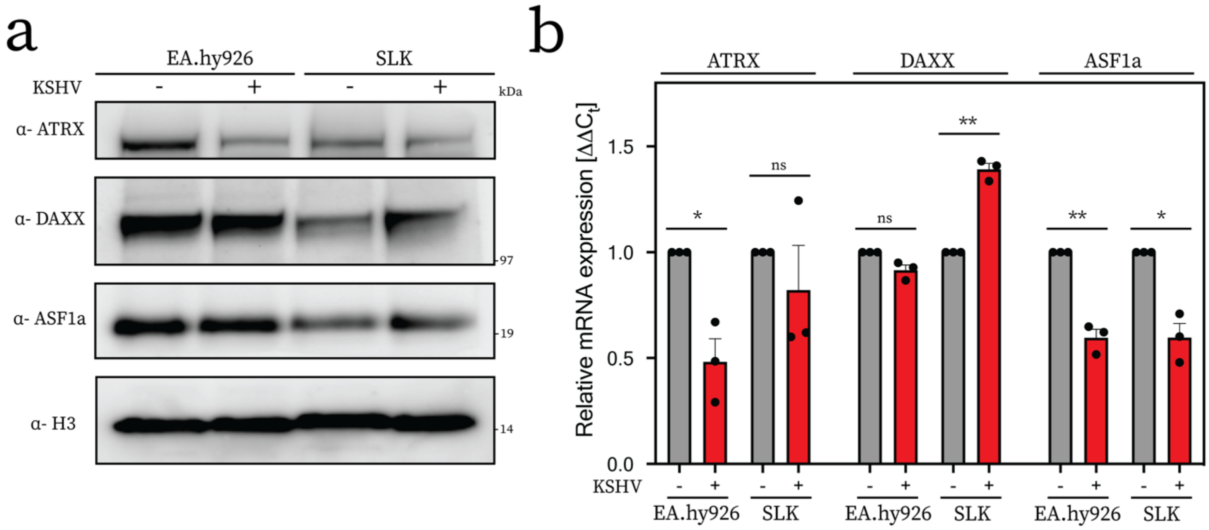

Supplementary figure 9: Analysis of factors involved in ALT induction. a, Representative western blot analysis of key factors implicated in the induction of ALT by previous reports. Experiment was performed in at least in biological triplicate with similar results. **b,** Analysis of the expression of indicated transcripts by RT-qPCR. Data is normalised to signal obtained cDNA derived from RNA of uninfected cells respectively. Experiment was performed in technical triplicate, each dot represents a biological replicate of the experiment. Significance was tested by paired, two-tailed t-test (p-values from left to right: *0.0418, ^{ns}0.4863, ^{ns}0.0761, **0.0052, **0.0096, *0.0261). Error bars represent SEM.

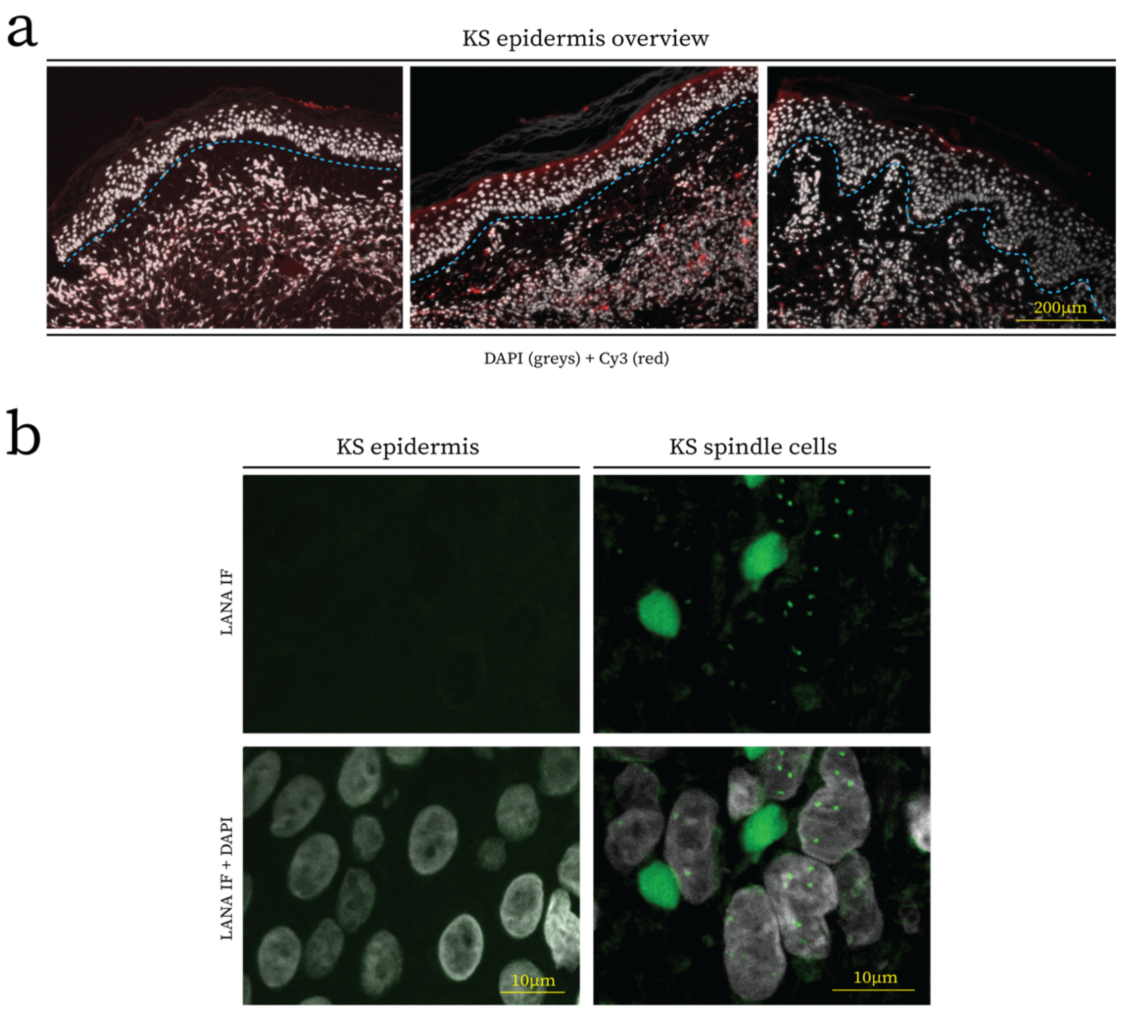

Supplementary figure 10: Overview of epidermal bystander cells in dermal KS. a, Identification of epidermis at margin of KS section. Images of the epidermis of three representative patients are shown. DAPI is shown in grey, autofluorescence of erythrocytes and extra-cellular matrix components is detected in Cy3 and shown in red. Dotted light blue line indicates lower border of epidermis as judged during image acquisition. **b,** LANA staining of epidermal bystander and KS spindle cells. Representative images generated by maximum intensity projection in Z of confocal micrographs are shown. LANA positive tumour cells and uninfected, LANA negative bystander cells are shown respectively. Representative of all patients included in analysis.

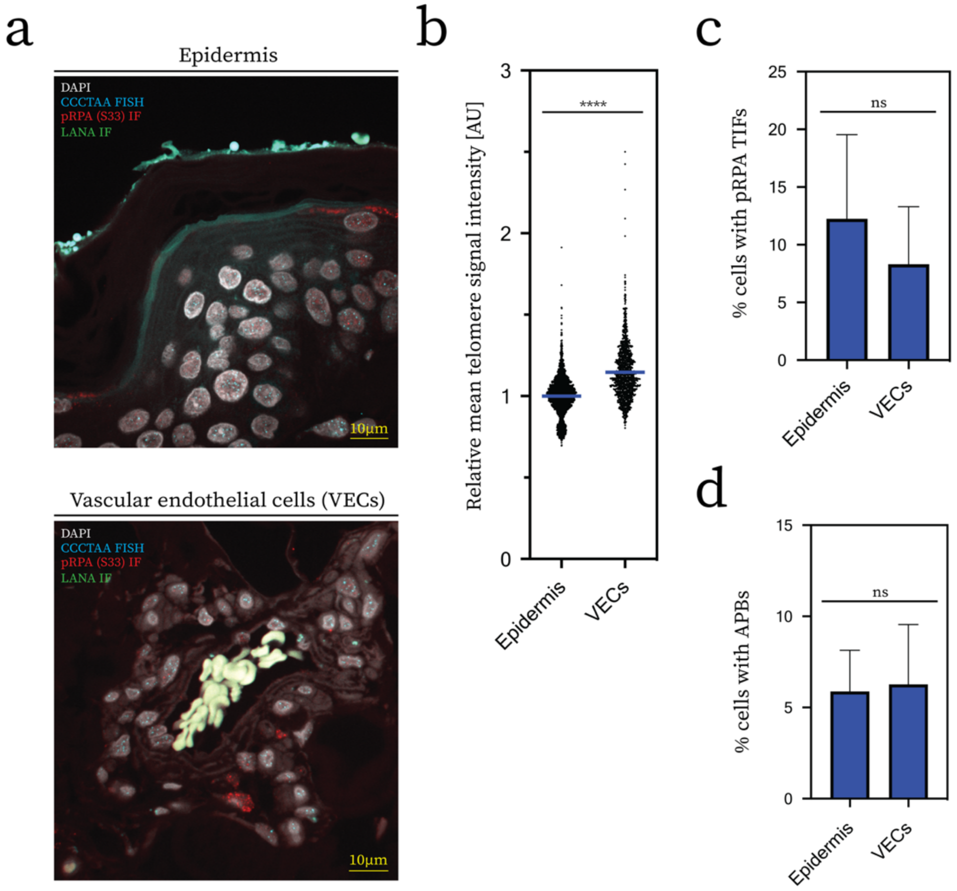

Supplementary figure 11: Healthy skin tissue controls for IHC/FISH analysis of dermal KS. a, Representative images of cell types included in control analysis obtained by maximum intensity projection of confocal micrographs in Z. Vascular endothelial cells (VECs) were used as approximate healthy equivalent of KS spindle cells (bottom image), whereas epidermal cells were identified as in analysis of tumour tissue (top image). **b,** Quantification of telomere signal intensity in normal cells. Images were obtained from VECs and epidermal cells within same section of healthy tissue derived from three individuals. **c,** Quantification of the proportion of cells with pRPA TIFs as described for tumour tissue (at least one pRPA/telomere co-localisation event). For both analyses presented in **(b,c)**, data representative of at least 30 nuclei per individual and cell type, statistical significance was tested by unpaired student's t-test (****$p<0.0001$, $^{ns}p>0.05$). Error bars represent SEM.

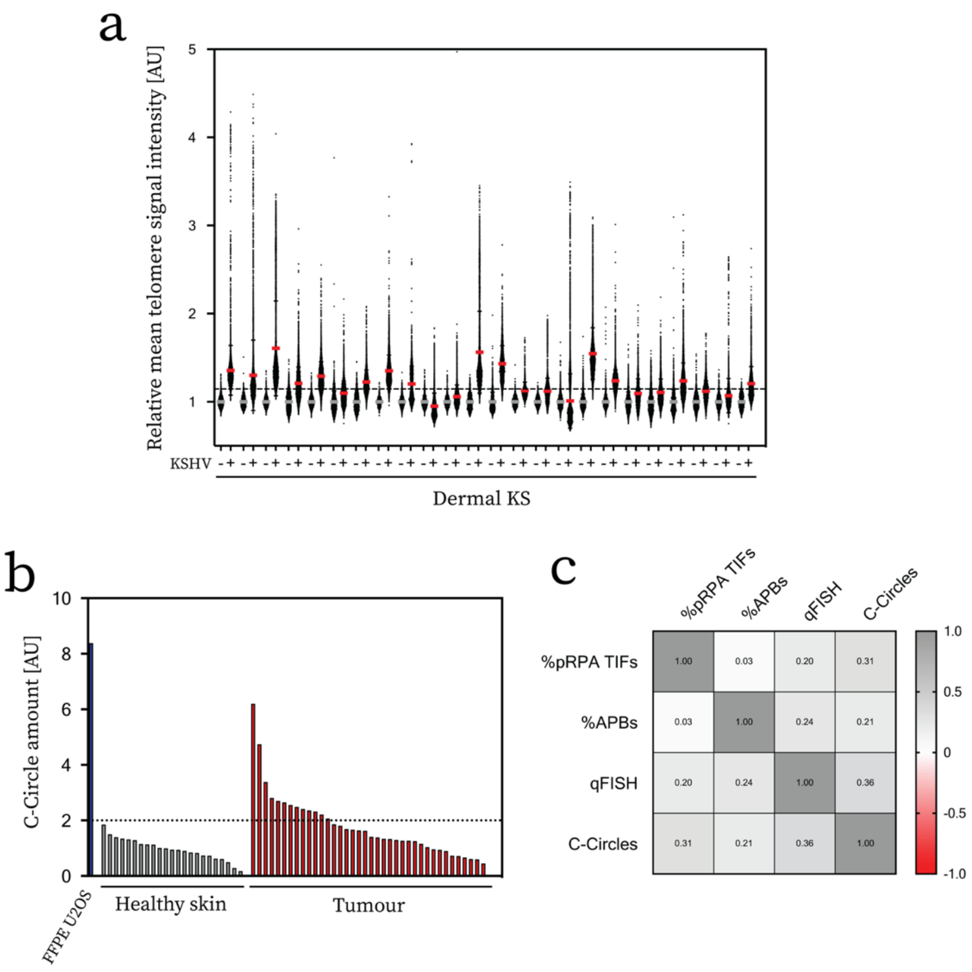

Supplementary figure 12: Examination of inter-patient variability in analysis of dermal KS for hallmarks of ALT relative to healthy skin tissue. a, Quantification of telomere signal intensity for individual patients. Telomere FISH signals of nuclei in KS spindle cells (red, +) were compared to bystander epidermal cells (green, -) from the same section. Data expressed as dot blot from at least 100 cells per patient, bars indicate mean. Each value within pairs of tumour and epidermal cells is normalised to the mean signal intensity of epidermal bystanders. Dotted line represents threshold as determined by analysis of healthy tissue, patients where mean difference surpassed this threshold were deemed positive (difference >1.145). **b,** Quantification of C-circle amount for individual patients. Each bar represents analysis of DNA obtained from one distinct tumour. Signal was normalised to Alu repeat loading control blot. FFPE U2OS (blue) and healthy control skin tissue (grey) are reported as positive and negative control respectively, details in Materials & Methods. Dotted line represents arbitrary threshold >2, in analogy to published reports, which was used to determine positive samples. **c,** Summary correlation matrix displaying Pearson's correlation coefficient for each of the ALT features examined in this study. Only patients where all measurements were obtained are considered in this analysis.

Supplementary table

-

KSHV *OriA* (qPCR and ddPCR)	Forward: 5’-CAAGCACGCGCATATAACCC 3’<br>Reverse: 5’-GGGATATGCTTCCGCCTCAT 3’<br>TaqMan probe: 5’ AGAAGCACAGCCACGCGTCA 3’.
RPP30 (ddPCR)	Forward: 5’ AGATTTGGACCTGCGAGC 3’<br>Reverse: 5’ ATCAGCCTCCAGGGACAT 3’<br>TaqMan probe: 5’ CTGACCTGAAGGCTCTGCGC 3’
GAPDH (RT-qPCR)	Forward: 5’ GTCTCCTCTGACTTCAACAGCG 3’<br>Reverse: 5’ ACCACCCTGTTGCTGTAGCCAA 3’
hTERT (RT-qPCR)	Forward: 5’ CGGAAGAGTGTCTGGAGCAA 3’<br>Reverse: 5’ GGATGAAGCGGAGTCTGGA 3’
ATRX (RT-qPCR)	Forward: 5’ AGAAATTGAGGATGCTTCACC 3’<br>Reverse: 5’ TGAACCTGGGGACTTCTTTG 3’
DAXX (RT-qPCR)	Forward: 5’ TGCAGACACCCCCGAAGCCT 3’<br>Reverse: 5’ TGCCATTCCACTAGGGCCCTCA 3’
ASF1a (RT-qPCR)	Forward: 5’ CAGATGCAGATGCAGTAGGC 3’<br>Reverse: 5’ CCTGGGATTAGATGCCAAAA 3’
Telomere primers (TRF SB)	Forward: 5’ TTAGGGTTAGGGTTAGGGTTAGGG 3’<br>Reverse: 5’ AATCCCAATCCCAATCCCAATCCC 3’
Telomere probe (C-Circle assay)	5’ CCCTAACCCTAACCCTAA 3’
Alu probe (C-Circle assay)	5’ GTAATCCCAGCACTTTGG 3’

Supplementary table 1: Primers and oligonucleotides used in the study
