# Supplementary material for: Oncogenic herpesvirus KSHV triggers hallmarks of alternative lengthening of telomeres
Source: Nat Commun. 2021 Jan 21;12:512. doi: 10.1038/s41467-020-20819-4 (PMC7820467; doi:10.1038/s41467-020-20819-4)
Supplement: Supplementary file 3 — Reporting Summary [file 41467_2020_20819_MOESM3_ESM.pdf]

nature research

Corresponding author(s): Simon Boulton

Last updated by author(s): Nov 30, 2020

Reporting Summary

Nature Research wishes to improve the reproducibility of the work that we publish. This form provides structure for consistency and transparency in reporting. For further information on Nature Research policies, see our Editorial Policies and the Editorial Policy Checklist.

Statistics

For all statistical analyses, confirm that the following items are present in the figure legend, table legend, main text, or Methods section.

n/a	Confirmed	
☐	☒	The exact sample size (*n*) for each experimental group/condition, given as a discrete number and unit of measurement
☐	☒	A statement on whether measurements were taken from distinct samples or whether the same sample was measured repeatedly
☐	☒	The statistical test(s) used AND whether they are one- or two-sided<br>*Only common tests should be described solely by name; describe more complex techniques in the Methods section.*
☒	☐	A description of all covariates tested
☐	☒	A description of any assumptions or corrections, such as tests of normality and adjustment for multiple comparisons
☐	☒	A full description of the statistical parameters including central tendency (e.g. means) or other basic estimates (e.g. regression coefficient) AND variation (e.g. standard deviation) or associated estimates of uncertainty (e.g. confidence intervals)
☐	☒	For null hypothesis testing, the test statistic (e.g. *F*, *t*, *r*) with confidence intervals, effect sizes, degrees of freedom and *P* value noted<br>*Give P values as exact values whenever suitable.*
☒	☐	For Bayesian analysis, information on the choice of priors and Markov chain Monte Carlo settings
☒	☐	For hierarchical and complex designs, identification of the appropriate level for tests and full reporting of outcomes
☐	☒	Estimates of effect sizes (e.g. Cohen's *d*, Pearson's *r*), indicating how they were calculated

Our web collection on statistics for biologists contains articles on many of the points above.

Software and code

Policy information about availability of computer code

Data collection	BD FACS Diva 6, Micromanager 1.2, Nikon NIS Elements Advanced Research
Data analysis	CellProfiler 2, FIJI 1, FlowJo 10

For manuscripts utilizing custom algorithms or software that are central to the research but not yet described in published literature, software must be made available to editors and reviewers. We strongly encourage code deposition in a community repository (e.g. GitHub). See the Nature Research guidelines for submitting code & software for further information.

Data

Policy information about availability of data

All manuscripts must include a data availability statement. This statement should provide the following information, where applicable:

- Accession codes, unique identifiers, or web links for publicly available datasets
- A list of figures that have associated raw data
- A description of any restrictions on data availability

The datasets generated during and/or analysed during the current study are available from the corresponding author on reasonable request.

Field-specific reporting

Please select the one below that is the best fit for your research. If you are not sure, read the appropriate sections before making your selection.

☒ Life sciences ☐ Behavioural & social sciences ☐ Ecological, evolutionary & environmental sciences

For a reference copy of the document with all sections, see nature.com/documents/nr-reporting-summary-flat.pdf

Life sciences study design

All studies must disclose on these points even when the disclosure is negative.

Sample size	All cell lines were established at least three times independently to exclude any effects due to clonal selection. Cell biology analysis in vitro was performed using sample size established in the field (n=50 for each experiment). By default, all data points were included in automated image analysis by CellProfiler. Sample size for analysis of primary tumours was determined by the availability of patient material.
Data exclusions	All data was included in the analysis presented here with the exception of: Microscopy images of poor quality due to acquisition errors and clear outliers within technical replicates of qPCR analysis.
Replication	All experiments were performed in at least biological triplicate with similar results, except for PICh/mass spectrometry, which was performed once. In addition to this, experiments where manual pipetting error is a possibility (qPCR, multiwell clonogenic assay), three technical replicates were performed for each condition.
Randomization	Randomization was not applicable to any of the experimental procedures in this study.
Blinding	Data sets were blinded for manual scoring of microscopy images using a custom Bash script, kindly provided by Dr Mark Robertson (Feldhahn group).

Reporting for specific materials, systems and methods

We require information from authors about some types of materials, experimental systems and methods used in many studies. Here, indicate whether each material, system or method listed is relevant to your study. If you are not sure if a list item applies to your research, read the appropriate section before selecting a response.

Materials & experimental systems

n/a	Involved in the study	
☐	☒	Antibodies
☐	☒	Eukaryotic cell lines
☒	☐	Palaeontology and archaeology
☒	☐	Animals and other organisms
☐	☒	Human research participants
☒	☐	Clinical data
☒	☐	Dual use research of concern

Methods

n/a	Involved in the study	
☒	☐	ChIP-seq
☐	☒	Flow cytometry
☒	☐	MRI-based neuroimaging

Antibodies

Antibodies used	α-RAD52 (Abcam ab124971) WB 1:2,000, α-BLM (Abcam ab2179) WB 1:1,000, α-PolD3 (Abcam ab182564) WB 1:5,000, α-SLX4 (kind gift by Prof. John Rousse) WB 1:5,000, α-ATRX (Bethyl A301-045A) WB 1:500, α-DAXX (Santa Cruz sc7152) WB 1;1,000, α-H3 (Abcam ab10799) WB 1:2,000, α-ASF1a (Cell Signalling 2990) WB 1:1,000, α-LANA (Millipore MABE1109) WB 1:1,000, IF 1:250. α-ORF57 (Santa Cruz sc135746) WB 1:1,000, α-beta actin (Biolegend 664802) WB 1;2,000. HRP-coupled secondary antibodies α-mouse (Dako P0447) WB 1:2,000, α-rabbit (Dako P0399) WB 1:2,000, α-rat (Abcam ab97057) WB 1:2,000, α-PML (Santa Cruz sc966) IF 1:500, α-RPA (phosphorylated on S33, Bethyl A300-246A) IF 1:2,000, α-mouse-Alexa647 (Invitrogen A21236) IF 1:500, α-rat-Alexa488 (Invitrogen A1106) IF 1:500, α-rabbit-647 (Invitrogen A21245) IF 1:500, α-BrdU (GE RPN202) WB at dilution supplied by manufacturer (part of a kit), α-BrdU (BD Biosciences #347580) BrdU IP assay: 0.5ug per pulldown.
Validation	α-SLX4 antibody was validated as described in Panier et al., 2019 (https://doi.org/10.1016/j.molcel.2019.07.010). All other antibodies are commercially available and further information may be found on the respective manufacturer's website using the identification numbers provided here. In addition to this, α-ATRX band specificity was validated in the Boulton lab by analysis of ATRX knockout cell lines. RAD52, PolD3, BLM antibody specificity was further validated by RNAi. LANA antibody specificity was concluded by strong staining in KSHV-infected cells, which was absent in uninfected counterparts.

Eukaryotic cell lines

Policy information about cell lines

Cell line source(s)	SLK, iSLK.219, and BJAB cells were obtained from Dr Grzegorz Sarek; BJAB +rKSHV.219 and uninfected BJAB cells were obtained from Prof. Thomas Schulz, Hanover Medical School. EA.hy926 cells were obtained from ATCC.
Authentication	Cell lines were authenticated by STR profiling performed by the Francis Crick Institute Cell Services Science & Technology Platform (STP).
Mycoplasma contamination	Cell lines were found to be negative for mycoplasma contamination throughout. This was determined routinely by PCR performed by the studies first author as well as Crick Cell Services STP using multiple methods (PCR, IF, soft agar).
Commonly misidentified lines (See ICLAC register)	SLK is commonly misidentified (de facto Caki1 celar cell renal carcinoma cell line). Initially, it was thought to represent the KS cell of origin, eg. vascular endothelium. We initially used SLK in our study to ensure comparability with the many reports in the KSHV field, which utilise this cell line. However, we later also included EA.hy926 as a "true" endothelium-like cell line in our study.

Human research participants

Policy information about studies involving human research participants

Population characteristics	The KS cases included in this study were mostly HIV positive (72%) with some HIV negative cases (28%). All tumours analysed were dermal KS biopsies. Participants were aged 23-75 years at the time of biopsy (median 46; SD 13.95). All cases included in the study were dermal KS.
Recruitment	Patients were selected by Prof. Mark Bower. They comprise the majority of KS patients seen in London hospitals during a fixed time period of three years. All patients seen during this time period by the investigator have been included in this study and at the time of selection, the investigation present in this paper was not underway. Therefore, we are confident that there is no selection bias.
Ethics oversight	Imperial College London, Human Tissue Act (details in methods section, paragraph 1).

Note that full information on the approval of the study protocol must also be provided in the manuscript.

Flow Cytometry

Plots

Confirm that:

☒ The axis labels state the marker and fluorochrome used (e.g. CD4-FITC).

☒ The axis scales are clearly visible. Include numbers along axes only for bottom left plot of group (a 'group' is an analysis of identical markers).

☒ All plots are contour plots with outliers or pseudocolor plots.

☒ A numerical value for number of cells or percentage (with statistics) is provided.

Methodology

Sample preparation	Cells were fixed with Formaldehyde and ice-cold Methanol (see full description in methods section).
Instrument	BD FACS Aria
Software	BD FACSDiva
Cell population abundance	Abundance of cells corresponding to each cell cycle stage was determined by equal application of gates (see below) and division with the total cell number analyzed for each sample respectively.
Gating strategy	Cell cycle stages were gated in agreement with previously published reports since the staining obtained was near identical ot the profile detected in other cell lines. Specifically, we denoted cells with EdU high as S-phase, EdU low/DAPI low as G1-phase, and EdU low/DAPI high as G2-phase cells respectively.

☒ Tick this box to confirm that a figure exemplifying the gating strategy is provided in the Supplementary Information.
